# Targeting Cancer Cell Energy Metabolism in Colorectal Cancer: Opportunities and Challenges from Drug Repositioning

**DOI:** 10.3390/cells14241968

**Published:** 2025-12-11

**Authors:** Lorenzo Tomassini, Teresa Pacifico, Giovanni Monteleone, Carmine Stolfi, Federica Laudisi

**Affiliations:** 1 Department of Systems Medicine, University of Rome “Tor Vergata”, 00133 Rome, Italyteresa.pacifico@uniroma2.it (T.P.); gi.monteleone@med.uniroma2.it (G.M.); carmine.stolfi@uniroma2.it (C.S.); 2 Gastroenterology Unit, Policlinico Universitario Tor Vergata, 00133 Rome, Italy

**Keywords:** cancer metabolism, Warburg effect, mitochondria, drug repurposing, anti-diabetic drugs, cardiovascular drugs, nonsteroidal anti-inflammatory drugs, anthelmintic agents, antidepressants

## Abstract

**Highlights:**

**What are the main findings?**

- Multiple repurposed drugs act on metabolic vulnerabilities of colorectal cancer (CRC), revealing untapped therapeutic potential beyond their original indications.
- These agents target key metabolic and signaling pathways (e.g., AMPK/mTOR, glycolysis) to suppress CRC growth and improve response to standard therapies.

**What are the implications of the main findings?**

- Repositioning existing drugs provides a time- and cost-efficient route to accelerate therapeutic innovation in CRC.
- Modulating CRC metabolism through repurposed compounds supports precision medicine paradigms and the rational design of combination therapies.

**Abstract:**

Drug repositioning, also known as drug repurposing, represents a cost-effective and time-efficient approach to accelerate the development of novel therapies for colorectal cancer (CRC), the third most common cancer worldwide, with an estimated two million new cases and nearly one million deaths annually. This review aims to critically evaluate how existing non-oncologic drugs can be repositioned to exploit key metabolic vulnerabilities of CRC cells. Targeting cancer cell metabolism offers a unique therapeutic advantage, as it disrupts the bioenergetic and biosynthetic processes that sustain tumor growth, adaptation, and resistance to therapy. Specifically, we examine the mechanisms through which antidiabetic, cardiovascular, anti-inflammatory, antidepressant, and anthelmintic agents interfere with glycolysis, oxidative phosphorylation (OxPhos), and mitochondrial bioenergetics—metabolic circuits central to CRC progression and recurrence. By integrating recent mechanistic, preclinical, and clinical findings, we highlight how these repurposed drugs converge on major metabolic regulators, including the AMPK/mTOR signaling pathways, and how they can potentiate the efficacy of standard chemotherapies and immunotherapies. Furthermore, we discuss the translational challenges that must be addressed to move these compounds into clinical use. Collectively, this review underscores the therapeutic potential of targeting CRC metabolism through drug repositioning as a promising avenue toward more effective and personalized treatment strategies.

## 1. Introduction

Colorectal cancer (CRC) is the second leading cause of cancer-related mortality and the third most commonly diagnosed malignancy worldwide [1]. Advances in both primary and adjuvant treatments have significantly improved patient survival over the last decades. The primary therapeutic goal remains the complete removal of the tumor, which typically requires surgical intervention. Although widespread screening programs aim to lower the incidence and facilitate early detection, about 25% of CRC cases are still diagnosed at an advanced stage with distant metastasis. Many of these patients later develop metachronous metastatic disease, which further raises the risk of death [2]. For patients with unresectable disease or who are not surgical candidates, treatments focus on reducing tumor size and preventing further progression. In these cases, chemotherapy and radiotherapy are the primary options and can also be used before or after surgery to maximize tumor shrinkage and stabilize the disease. However, a significant number of patients can experience resistance to cancer drugs, either at the beginning or during treatment, which eventually results in therapy failure. Immune-checkpoint-targeted therapies aim to enhance immune surveillance and cytotoxic activity against cancer cells by inhibiting tumor-mediated evasion of T-cell detection [3]. Checkpoint inhibitors have demonstrated encouraging therapeutic responses across a range of solid malignancies, including CRC [4]. In metastatic CRC, early-phase clinical trials showed notable responses in select cases, but only a small group of them benefit from immune checkpoint blockade, specifically those with high tumor mutational burden (TMB) and tumors exhibiting microsatellite instability-high (MSI-H) or mismatch repair-deficient (dMMR) phenotypes [5,6]. These issues require alternative therapeutic options and are prompting researchers to explore new clinical approaches.

Another key aspect of CRC is its high heterogeneity, characterized by significant inter- and intra-tumoral variability that influence disease progression, treatment response, and patient outcomes. This complexity reflects the coexistence of diverse molecular and metabolic alterations both within and across tumors, encompassing differences in genetic mutations, epigenetic regulation, and tumor–microenvironment interactions. Importantly, tumor location along the colorectal axis significantly contributes to this complexity. Right-sided (proximal) and left-sided (distal) CRCs, as well as rectal cancers, exhibit distinct histological features, molecular signatures, metabolic profiles, and clinical behaviors that often guide therapeutic decision-making [7].

Proximal carcinomas tend to be mucinous, microsatellite unstable (MSI-high), and harbor mutations in key tumorigenic pathways. They also exhibit a B-Raf proto-oncogene, serine/threonine kinase (BRAF)-like signature and a serrated pathway profile, regardless of histological type [7,8,9]. In contrast, distal carcinomas were more often characterized by chromosome instability and amplification of EGFR or HER2, accompanied by increased epiregulin expression. These molecular distinctions are associated with differential responses to targeted therapies and chemotherapeutic agents [7,8,9]. Moreover, rectal cancer represents a clinically distinct entity due to its unique anatomical location, patterns of local invasion, and specific management strategies [10]. Most cases are conventional adenocarcinomas; however, mucinous and signet-ring cell subtypes, which are associated with poorer prognosis, occur more frequently than in colon tumors. At the molecular level, rectal cancers are typically defined by chromosomal instability and frequent mutations in *APC*, *KRAS*, *TP53*, and *PIK3CA*, with lower rates of *BRAF* mutations and CIMP positivity compared to right-sided colon cancers [10]. Transcriptomic profiles reveal similarities to left-sided tumors, including activation of Wnt/β-catenin and EGFR pathways, as well as genes promoting invasion and epithelial–mesenchymal transition [8,10].

Recognizing these spatial and molecular differences is crucial for understanding CRC biology and for developing therapeutic strategies that target shared metabolic and signaling vulnerabilities across heterogeneous CRC subtypes.

## 2. Cancer Energy Metabolism in CRC

In this context, cancer energy metabolism has emerged as a promising frontier for therapeutic innovation in CRC. Tumor cells undergo profound metabolic reprogramming to sustain uncontrolled growth, survival, and adaptation to stress, fundamentally altering the balance between major energy-generating pathways such as glycolysis and oxidative phosphorylation (OxPhos) [11,12]. This reprogramming supports not only ATP production but also the generation of biosynthetic precursors and redox cofactors required for macromolecule synthesis and maintenance of oxidative balance. The classic Warburg effect—whereby cancer cells preferentially utilize glycolysis for ATP generation even in the presence of oxygen—has long been considered a hallmark of tumor metabolism [13,14]. However, accumulating evidence indicates that many cancer types, including CRC, do not completely abandon mitochondrial respiration. Instead, they exhibit metabolic plasticity, dynamically switching between glycolysis and OxPhos in response to nutrient availability, oncogenic signaling, and microenvironmental cues [13,15,16,17].

Mitochondria are central players in this adaptable metabolic network. Beyond their canonical role in oxidative phosphorylation, they orchestrate redox regulation, lipid biosynthesis, amino acid metabolism, and apoptotic signaling [18]. In CRC, mitochondrial function remains highly active despite the presence of somatic mutations in mitochondrial DNA or nuclear-encoded mitochondrial genes. Mitochondrial DNA is critical for energy production and is more prone to damage than nuclear DNA due to its structure and limited repair capacity. Mitochondrial DNA mutations can promote cancer progression and metastasis by increasing ROS, stabilizing HIF1, and enhancing tumor aggressiveness [19]. These organelles sustain tumor progression by fueling anabolic pathways through the tricarboxylic acid (TCA) cycle, providing intermediates such as citrate, α-ketoglutarate, and succinate, which also serve as metabolic signals influencing epigenetic and transcriptional regulation [20,21]. Importantly, disrupting mitochondrial activity in CRC has been shown to impair ATP production, disturb redox homeostasis, and trigger apoptosis or ferroptosis, thereby sensitizing tumor cells to chemotherapy, radiotherapy, and targeted agents [22]. Thus, strategies that target key components of mitochondrial metabolism—such as complex I inhibitors, mitochondrial pyruvate carriers, or regulators of fatty acid oxidation—are currently under investigation as means to exploit the metabolic vulnerabilities of CRC cells [22,23,24].

Finally, targeting the unique metabolic dependencies of CRC may help overcome resistance mechanisms and improve patient outcomes, although these dependencies can vary substantially across different CRC phenotypes and even within individual tumors. Continued investigation into cancer metabolism holds the potential to refine therapeutic strategies and foster the development of alternative, personalized treatments tailored to this heterogeneity.

## 3. Drug Repositioning to Target CRC Energy Metabolism

**Figure 1:**
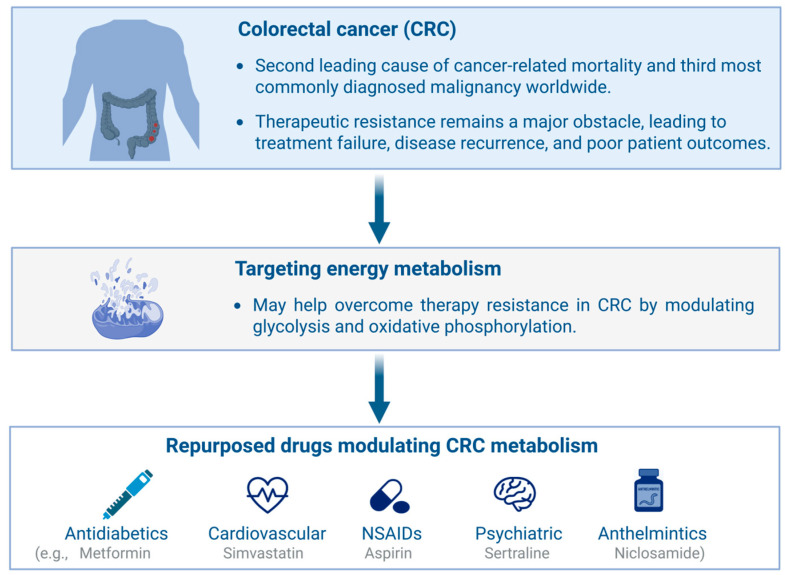
**Overview of drug repurposing strategies targeting metabolic vulnerabilities in colorectal cancer (CRC).** Altered glycolysis and oxidative phosphorylation contribute to therapy resistance, which can be counteracted by repurposed antidiabetic, cardiovascular, psychiatric, and anthelmintic drugs, as well as by nonsteroidal anti-inflammatory drugs (NSAIDs). Created in BioRender. https://BioRender.com/7063tnu (accessed on 23 November 2025).

Drug repositioning, also known as drug repurposing, refers to identifying new therapeutic applications for existing drugs, whether already approved or still under investigation, beyond their original medical indications [25]. This strategy has recently gained considerable attention as it can substantially reduce the time, cost, and risk associated with conventional drug discovery and development. By leveraging existing pharmacological and toxicological data, drug repositioning streamlines the regulatory process and facilitates the rapid advancement of promising compounds toward clinical application and eventual commercialization [26]. A major advantage of this approach lies in its reliance on well-established data regarding a drug’s absorption, distribution, metabolism, excretion, and toxicity (ADMET) properties—absorption, distribution, metabolism, excretion, and toxicity. These pharmacokinetic and safety profiles are typically well-characterized, especially for compounds that have advanced through or completed Phase III clinical trials. As such, these agents already demonstrate favorable safety and efficacy in large patient populations, significantly reducing the need for extensive early-stage laboratory testing and preclinical validation [27]. This accelerates development timelines and boosts clinical trial success rates by minimizing uncertainties regarding a drug’s pharmacokinetic and pharmacodynamic behavior. Consequently, drug repositioning is emerging as a strategic focus for pharmaceutical and biotech companies seeking to maximize the value of existing drug libraries while minimizing financial risk. Given the central role of energy metabolism in cancer development and progression, several drugs originally developed for metabolic disorders—such as anti-diabetic and cardiovascular agents—have been successfully repurposed for oncologic applications [28]. Furthermore, accumulating evidence highlights nonsteroidal anti-inflammatory drugs (NSAIDs), anthelmintic agents, and antidepressants as promising classes of compounds with potential anticancer activity through modulation of tumor energy metabolism and stress-response pathways (Figure 1).

### 3.1. Methodology

A literature review was conducted in PubMed up to May 2025 to identify studies investigating the interplay between CRC, cellular and mitochondrial metabolism, and the therapeutic effects of repurposed drugs. The search strategy combined disease-related, mechanistic, and drug-class terminology. Specifically, the term “*colorectal cancer*” was paired with “*metabolic reprogramming*,” “*mitochondrial metabolism*,” “*metabolism*,” “*tumor microenvironment*,” “*drug repositioning*,” and “*drug repurposing*,” as well as with keywords referring to major pharmacological classes explored for repurposing in oncology, including “*antidiabetic drugs*,” “*cardiovascular drugs*,” “*NSAIDs*,” “*antidepressants*,” and “*anthelmintics*.”

In addition to the pharmacological classes ultimately included in this review, the search strategy initially considered other categories commonly explored in oncology drug repurposing, such as antimalarial, antimicrobial, antiviral, and antiepileptic agents. However, these were not incorporated into the main discussion because evidence directly linking them to CRC-specific metabolic reprogramming was limited, inconsistent, or not mechanistically informative. Therefore, only drug classes with sufficiently robust and reproducible data on CRC metabolic or mitochondrial pathways were retained.

Eligible studies were peer-reviewed, published in English, and provided preclinical or clinical evidence linking repurposed pharmacological agents to CRC metabolism, mitochondrial function, or tumor–microenvironment modulation. We included in vitro and in vivo experimental studies, observational clinical research, and registered clinical trials. We excluded non-original articles, incomplete conference abstracts, and studies lacking mechanistic or functional metabolic endpoints. Reference lists of relevant articles were also screened manually to identify additional eligible studies.

The effects of pharmacological agents repurposed to target CRC cell metabolism are summarized in Table 1 and discussed below.

### 3.2. Anti-Diabetic Drugs

Among the various classes of anti-diabetic medications, metformin is the most extensively studied agent regarding its effects on carcinogenesis. Several epidemiological studies suggest that metformin may have a role in the chemoprevention of CRC due to an inverse association between long-term use of metformin and CRC risk. Moreover, substantial preclinical evidence has demonstrated metformin’s ability to inhibit tumor growth across many types of cancers, including CRC, by activating the AMP-activated protein kinase (AMPK), a key regulator of cellular energy balance [29]. AMPK affects not only glucose and lipid metabolism but also tumor bioenergetics by blocking the mammalian target of rapamycin (mTOR) pathway, which is critical for cell growth and division [30,31]. Beyond AMPK activation, metformin has demonstrated anti-cancer activity through additional mechanisms. For example, it has been shown to modulate inflammatory responses and promote cell death in cancer stem cells [32]. Notably, the inhibitory effects of metformin on CRC have been reported in vitro, where metformin-treated CRC cells expressed several miRNAs and mRNAs involved in cell metabolism and PI3K-Akt–MAPK/ERK signaling pathways [33]. Additional studies confirmed that most of the metformin-sensitizing miRNAs in CRC cells (HCT116 cells) were involved in regulating glycolysis or mitochondrial respiration [34]. Meanwhile, others reported a metformin-driven cytotoxic effect on butyrate-resistant PMF-K014 CRC spheroid cells through AMPK- and Akt-mediated mechanisms [35]. Moreover, metformin also demonstrated anti-tumor effects in preclinical models, including decreased spontaneous intestinal polyp formation in Apc^min/+^ mice and in a mouse model of sporadic CRC by enhancing AMPK activity [36,37]. It can also support anti-tumor immune responses in CRC patients by decreasing the production of neutrophil extracellular traps (NETs) released from tumor-associated neutrophils (TANs), thereby allowing the CD3^+^CD8^+^ lymphocytes to infiltrate the tumor [38].

Dapagliflozin is a sodium-glucose cotransporter-2 (SGLT2) inhibitor, a class of anti-diabetic drugs that limit renal glucose reabsorption by inhibiting SGLT2 receptors in the renal tubules. SGLT2 receptors are highly expressed by cancer cells compared to healthy human tissues, due to a greater demand for glucose for glycolysis and energy production, thus making them a valuable target for cancer treatment. Evidence for dapagliflozin’s anti-tumor effects in CRC is limited. Notably, it was shown to inhibit the growth of the MC38 CRC cell line in an obese mouse model, acting through an insulin-dependent pathway [39]. Similar outcomes were seen in HCT116 cells treated with dapagliflozin via a mechanism independent of SGLT2 inhibition, but involving ERK phosphorylation [40]. In contrast, the scenario is significantly different regarding the use of DPP4 inhibitors, a class of anti-diabetic drugs that target the dipeptidyl peptidase-4 (DPP4), also known as adenosine deaminase complexing protein 2 or CD26, which is involved in various physiological and pathological processes by regulating energy metabolism, inflammation, and immune function. DPP4 inhibitors have garnered significant interest regarding the treatment of various cancer types, including CRC. In particular, the compound sitagliptin has demonstrated potential anti-tumor and anti-metastatic properties in CRC. For example, Varela-Calviño R. and colleagues demonstrated that the invasive and migratory features of CD26^+^ CRC stem cells are significantly reduced upon treatment with sitagliptin [41]. Moreover, sitagliptin was reported to strongly bind, forming a stable complex, and negatively regulate the CD24/CTNNB1/SOX4 axis, which was previously demonstrated to promote CRC progression [42]. Interestingly, in vitro observations showed that sitagliptin sensitizes CRC cells to 5-Fluorouracil (5-FU) by decreasing the expression of MDR1 mRNA and the p-AKT and NFκB2 subunits p100/p52 proteins [43]. These findings suggest that DPP4 inhibitors could be a promising anti-tumor treatment for CRC, but further research is necessary to fully explore their therapeutic potential.

### 3.3. Cardiovascular Drugs

Statins function as competitive inhibitors of HMG-CoA reductase, the enzyme that controls the rate-limiting step in the mevalonate pathway. By blocking this pathway, statins decrease endogenous cholesterol production, resulting in lower circulating low-density lipoprotein (LDL) cholesterol. Aside from lowering lipids, statins have various pleiotropic effects, including anti-tumor actions through interference with cell metabolism. For example, studies have shown that lovastatin can increase the vulnerability of CRC cells to chemotherapy-induced apoptosis or cause mitochondrial dysfunction and energy depletion in HCT116 cells, which leads to mtDNA release and apoptosis via the cGAS-STING pathway [44,45]. Additional statins, including pitavastatin, have demonstrated anti-tumor effects in vivo using a mouse model of obesity-related CRC and APC^min/+^ mice by decreasing pro-inflammatory cytokine production and activating AMPK and oxidative stress in the colonic mucosa of AOM-treated mice [46]. Moreover, pitavastatin, together with the other statin atorvastatin, could inhibit cell proliferation and 3D spheroid formation of the CRC cell line SW480 under high glucose conditions [47]. In particular, they can both synergistically induce apoptosis by promoting 5-FU-mediated cytotoxic effect through autophagy activation, as well as the PERK/ATF4/CHOP signaling pathway, while decreasing YAP expression [47]. Conversely, simvastatin has been reported to induce apoptosis in the human CRC cell line HT-29 and suppress Akt activation by downregulating IGF-1R expression and promoting proapoptotic ERK signaling [48]. Collectively, these findings highlight the potential of statins not only as lipid-lowering agents but also as adjuvant or combination therapeutics in CRC treatment, by modulating metabolic reprogramming and inflammation-driven progression.

Cardiac glycosides (CGs) are organic compounds commonly used to treat heart failure and arrhythmias because they enhance the strength and rate of heart contractions. They work by binding to the extracellular sites of the Na^+^, K^+^-ATPase pump, a key membrane protein that maintains sodium and potassium ion gradients across the cell membrane. Present in various plants and amphibians, CGs inhibit the sodium-potassium ATPase, leading to a positive inotropic effect. In addition to their cardiovascular applications, recent studies have explored the anticancer potential of CGs, particularly oleandrin, which has attracted attention as a potential cancer therapy, although findings remain under debate. In particular, Pan L. and colleagues reported that oleandrin induces apoptosis in the CRC cell lines SW480, HCT116, and RKO by compromising mitochondrial activity [49]. Indeed, it promotes caspase-3 and caspase-9 activities, cytochrome c and BAX protein expressions, and intracellular calcium levels, while decreasing Bcl-2 and GSH levels [49]. Another study indicates that the Na^+^, K^+^-ATPase α3 subunit, which serves as a receptor for cardiac glycosides including oleandrin, was differentially expressed in cancer and normal lung and colon tissue, and this strongly influences anti-tumorigenic oleandrin effects [50]. In particular, the Na^+^, K^+^-ATPase α3 isoform was mainly located near the cytoplasmic membrane in normal human colon and lung epithelia, while its expression shifted to a peri-nuclear position in paired cancer cells [50]. Similarly, the distribution of the α3 isoform moved from the cytoplasmic membrane in differentiated human colon cancer CaCO-2 cells to a peri-nuclear area in undifferentiated CaCO-2 cells. Moreover, oleandrin showed a stronger anti-proliferative effect in undifferentiated than in differentiated cells, triggering autophagy and modulating ERK phosphorylation [50], making it an interesting compound to be exploited in cancer therapy.

Beta-adrenergic blocking agents (BBs), commonly known as β-blockers, are well-established cardiovascular drugs known for reducing mortality in hypertensive patients. Recently, there has been growing interest in repurposing β-blockers for cancer treatment, particularly in CRC. Some findings highlight their potential to suppress tumor growth and enhance therapeutic responses (e.g., chemotherapy or immunotherapy). CRC cells express β-adrenergic receptors (β-ARs), and their stimulation by stress hormones like adrenaline and noradrenaline has been shown to promote tumor proliferation and progression. Recent studies have focused on the therapeutic potential of β-blockers—especially those targeting β2-ARs—as adjuncts in CRC treatment. Experiments using HT-29 colon cancer cells demonstrated that β-AR agonists induce proliferation, whereas β-blockers can reverse these effects, significantly reducing cell growth. For example, the β2-AR selective blockade with ICI-118,551 has shown particular promise in enhancing radiation-induced apoptosis in CRC cells, especially those with functional p53, by promoting mitochondrial dysfunction and cytochrome c release and inhibiting EGFR–Akt–ERK1/2 signaling pathways [51]. Moreover, non-selective β-blocker propranolol decreased the ability of CRC cells to adapt to hypoxia [52]. Interestingly, the inhibition of adrenoreceptors by propranolol enhanced apoptosis, decreased the number of mitochondria, and lowered the amount of proteins involved in oxidative phosphorylation (i.e., V-ATP5A, IV-COX2, III-UQCRC2, II-SDHB, I-NDUFB8) [52].

### 3.4. Nonsteroidal Anti-Inflammatory Drugs (NSAIDs)

Non-steroidal anti-inflammatory drugs (NSAIDs) are commonly used for the treatment of inflammation and cardiovascular disease. Their primary mechanism involves inhibition of cyclooxygenase (COX) enzymes and prostaglandin E2 signaling, though several effects occur independently of COX. Apart from their ability to limit chronic inflammation in tumorigenesis, NSAIDs may also contribute to cancer treatment by triggering metabolic reprogramming. In particular, aspirin was seen to impact significantly on several enzymes and transporters involved in central carbon metabolism, resulting in reduced glutaminolysis following long-term treatment (52 weeks) [53]. Moreover, combination therapy treatment of Apc^fl/fl^ mice with the glutaminase 1 (GLS1) inhibitor CB-839 was able to impair crypt proliferation in the small intestine [53]. Aspirin was also reported to affect the activity of glucose-6-phosphate dehydrogenase (G6PD), which plays a crucial role in sustaining tumor growth [54]. Indeed, aspirin could acetylate and inhibit G6PD in HCT116 cells by targeting 14 lysine residues, including the catalytically essential K235 [54]. Interestingly, additional acetylated proteins by aspirin were identified in CRC cells, which are involved in the glycolytic pathways, that are aldolase, GAPDH, PGM, enolase, PKM2, LDH-A, and LDH-B [55]. Aspirin impacts cancer metabolism also by triggering AMPK activation, with the consequent inhibition of mTOR signaling. In particular, it promotes the expression of the miR-34a and miR-34b/c genes, which encode tumor-suppressive microRNAs, through a mechanism involving the activation of AMPK, which in turn stimulates NRF2, leading to direct induction of miR-34a/b/c expression via ARE motifs. Additionally, aspirin downregulates c-Myc, a known inhibitor of NRF2-driven transcription, through AMPK activation. Moreover, aspirin suppressed mTOR signaling in CRC cells through inhibition of the downstream effectors S6K1 and 4E-BP1 [56]. Indeed, treatment with aspirin altered nucleotide ratios and activated AMPK in CRC cells. However, mTOR inhibition persisted even after AMPKα knockdown by siRNA, suggesting that aspirin acts via both AMPK-dependent and AMPK-independent mechanisms [56]. Consistent with mTOR inhibition, aspirin promoted autophagy in CRC cells. Furthermore, combined treatment with aspirin and metformin enhanced suppression of mTOR and Akt while further stimulating autophagy. Finally, rectal mucosal samples from aspirin-treated patients exhibited reduced phosphorylation of S6K1 and S6 [56]. Aspirin was also reported to impact CRC metabolism in clinical studies. Results from an untargeted metabolomics approach identified 10,269 metabolic features in normal mucosal biopsies from 325 participants with a recent history of histologically confirmed adenomas who underwent colonoscopy after about three years of treatment with either a placebo or aspirin (81 or 325 mg/day) [57]. The authors identified 471 metabolic features in colon tissue associated with aspirin use compared to placebo. The metabolic pathways affected by aspirin differ by dose. For the 81 mg dose, the pathways involved pyrimidine metabolism, while for the 325 mg dose, they included arachidonic acid metabolism. The one pathway common to both doses was the carnitine shuttle. Among the aspirin-associated metabolites, three stood out as being associated also with adenoma risk, and these might mediate chemopreventive effects, i.e., creatinine, glycerol-3-phosphate and linoleate [57]. Glycerol-3-phosphate and linoleate are part of the glycerophospholipid metabolism pathway, which is relevant upstream of eicosanoid synthesis (which involves arachidonic acid and cyclooxygenase, the target of aspirin). Interestingly, carnitine shuttle metabolites (involved in fatty acid oxidation/transport into mitochondria) were increased by aspirin treatment, and higher levels of those metabolites were associated with increased adenoma risk [57]. Further investigations were conducted in 523 participants from the same clinical trial to characterize plasma metabolomics upon aspirin administration [58]. Changes in linoleate metabolism and glycerophospholipid metabolism were reported in patients receiving the 81 mg dose, while for both doses (81 mg and 325 mg), an altered carnitine shuttle pathway was observed [58]. Moreover, 81 mg/day aspirin treatment is associated with increased levels of lysophosphatidylcholines, lysophosphatidylethanolamines, and trihydroxyoctadecenoic acid (a derivative of linoleic acid upstream in the cyclooxygenase/arachidonic acid pathway), which is associated with decreased risk of adenomas during follow-up, thus confirming previous data in the colon tissue [58]. We can conclude that changes in glycerophospholipid metabolism suggest that aspirin is linked to a lower adenoma risk, even though higher levels of carnitine imply it might offset these benefits.

Beyond aspirin, other NSAIDs may exert anti-tumor effects on CRC cells through metabolic and stress-response modulation. Diclofenac, for example, reduces c-Myc protein expression, lactate dehydrogenase (LDH) activity, and the abundance of stress-related proteins including HSF1, Hsp70, and Hsp27, with concomitant loss of membrane Hsp70 positivity in CRC cells (i.e., LS174T, LoVo), but not in A549 lung, MDA-MB-231 breast, or COLO357 pancreatic carcinoma cells [59]. Diclofenac-mediated impairment of lactate metabolism and stress adaptation correlated with enhanced sensitivity to 5-FU and ionizing radiation in vitro, and radiosensitization in CRC xenografts in vivo, suggesting that suppression of LDH activity and/or stress responses underlies its sensitizing capacity [59]. In parallel, celecoxib synergizes with the thioredoxin reductase inhibitor auranofin, an FDA-approved antirheumatic agent, to amplify oxidative stress, disrupt mitochondrial redox homeostasis, inhibit hexokinase activity, and impair ATP generation, resulting in profound antitumor effects in vivo [60].

### 3.5. Antidepressants

Considerable attention has been directed toward the potential repurposing of antidepressants in cancer therapy. These drugs are often prescribed to cancer patients to manage depressive symptoms, and accumulating evidence suggests that some antidepressants may also exert direct anticancer effects or enhance the efficacy of conventional therapies. One emerging mechanism by which antidepressants may affect cancer biology involves the modulation of autophagy—a cellular self-digestion/recycling process implicated in cancer cell survival, death, metabolism and therapy resistance. In the context of CRC, selective serotonin reuptake inhibitors (SSRIs) have demonstrated, in preclinical models, the ability to interfere with multiple cancer hallmarks. These effects occur through coordinated alterations in metabolic reprogramming, mitochondrial and lysosomal function, autophagy, apoptosis, and cell-cycle regulation. Fluoxetine, one of the best-studied SSRI, exhibits multifaceted antitumor activity through a biphasic cell death mechanism. For example, Marcinkute and colleagues reported that fluoxetine induces p53-independent apoptosis, characterized by mitochondrial membrane depolarization and phosphatidylserine externalization [61]. This apoptotic process—evidenced by DNA fragmentation and an increase in sub-G1 and G0/G1 cell populations—is accompanied by a secondary necrotic pathway driven by mitochondrial calcium overload and ATP depletion [61]. The resulting disruption of calcium homeostasis within the endoplasmic reticulum markedly reduces cellular energy production, impairing both OxPhos and glycolytic flux [61]. In vivo, these metabolic perturbations translate into reduced xenograft growth, decreased angiogenesis, altered lactate metabolism, and diminished levels of mitochondrial complexes III and V, underscoring the profound impact of fluoxetine on CRC metabolic homeostasis [62,63]. By suppressing lactate export and modulating MCT4 expression, fluoxetine further destabilizes the tumor microenvironment, exacerbating metabolic stress and limiting cancer cell proliferation [63]. Complementing these effects, sertraline, another SSRI, exhibits anticancer activity by promoting autophagy and modulating apoptotic and cell-cycle pathways. In CRC cells, it synergistically enhances paclitaxel-induced autophagy by increasing autophagosome formation—as indicated by elevated LC3-II/LC3-I ratios and SQSTM1/p62 degradation [64]. This activation is accompanied by G0/G1 cell-cycle arrest, reactive oxygen species (ROS) accumulation, and regulation of apoptotic mediators, including downregulation of Bcl-2 and upregulation of c-Jun, while simultaneously sensitizing cells to chemotherapeutic agents such as 5-fluorouracil (5-FU) [65]. Other antidepressants display distinct yet convergent anticancer mechanisms. For instance, citalopram alters CRC cell metabolism, leading to reduced proliferation and increased apoptosis [66]. Vilazodone, in turn, enhances regorafenib sensitivity by promoting TRIM21-mediated selective autophagic degradation of c-Myc, thereby reducing ENO2 activity, suppressing glycolytic flux, and counteracting KRAS-driven metabolic reprogramming [67,68].

Collectively, psychotropic drugs are emerging as promising anticancer agents, offering a safe, economical, and clinically feasible avenue for therapeutic innovation. Their dual targeting of autophagy and mitochondrial metabolism—facilitated by their cationic amphiphilic structures—positions them as attractive candidates for future combinatorial strategies in CRC treatment.

### 3.6. Anthelmintic Drugs

Several anthelmintic drugs have demonstrated significant metabolic effects in CRC. Niclosamide, a salicylanilide traditionally used against tapeworms, functions as a mitochondrial uncoupler, collapsing the proton gradient to reduce ATP synthesis, elevating ROS, and triggering apoptosis in metabolically active cancer cells [69]. It also suppresses Wnt/β-catenin signaling via autophagic degradation and shifts tumor metabolism from the Warburg phenotype toward OxPhos by lowering lactate production and pentose phosphate pathway activity [70]. Similarly, oxyclozanide, a structural analog of niclosamide, disrupts mitochondrial OxPhos, inducing cancer cell death through bioenergetic collapse [71]. Nitazoxanide, a thiazolide, exerts anti-tumor effects by activating AMPK while inhibiting AKT/mTOR and Wnt/β-catenin signaling, thereby reducing proliferation and stemness—effects that are further enhanced when combined with farnesoid X receptor (FXR) agonists [72]. Benzimidazole derivatives, particularly flubendazole, have gained increasing attention for their anticancer properties beyond microtubule disruption. For example, flubendazole decreases p-mTOR, P62, Bcl-2, and STAT3 expression while inducing Beclin-1 and LC3 I/II, thereby triggering autophagy [73,74]. Pyrvinium pamoate, a quinoline-derived anthelmintic, has been identified as a potent metabolic inhibitor across various malignancies. This effect arises from its ability to target mitochondrial respiration and energy-sensing pathways critical for cancer cell metabolism. Unlike other anthelmintics, pyrvinium pamoate preferentially inhibits mitochondrial complex I and NADH-fumarate reductase activity, causing a collapse of mitochondrial respiration under hypoxic or glucose-deprived conditions [75,76]. This leads to ATP depletion, ROS accumulation, and disruption of redox balance, selectively impairing cancer cell survival within the tumor microenvironment. In CRC models, pyrvinium pamoate has not been reported to impact mitochondrial activity but rather to downregulate STAT3 phosphorylation, a key metabolic and oncogenic transcription factor, and to suppress PI3K/Akt/mTOR signaling, ultimately leading to cell cycle arrest and autophagy-mediated cell death [75].

## 4. Clinical Implications

Translating metabolic targeting into clinical practice has become an increasing priority in CRC research. A growing number of clinical trials are currently evaluating the efficacy of repurposed agents with well-established safety profiles (Table 2).

Among these compounds, metformin is the most extensively investigated, having been evaluated in multiple phase II and III trials either as a single agent or in combination with chemotherapy, radiotherapy, or immunotherapy (Table 2). Although numerous studies have been conducted, only a few have reported clinical outcomes, ranging from improved tumor regression following neo-adjuvant chemoradiotherapy in rectal cancer to a lack of objective response (NCT03800602, NCT03359681) [77]. Statins, particularly simvastatin, have also been tested as adjuvants to conventional regimens such as XELOX, where they did not increase toxicity, but showed comparable efficacy in patients with metastatic CRC as first-line chemotherapy [78], Cetuximab, where it was not able to restore sensitivity in CRC patients harbouring a somatic KRAS mutation [79], and FOLFIRI where, indeed, it was able to prolong time to progression in CRC patients [80]. Anti-inflammatory agents, such as aspirin and celecoxib, are currently being investigated in several phase II–IV clinical trials. However, published data remain limited and show inconsistent outcomes. A recent study (NCT04534218) suggested that combining multimodal metronomic chemotherapy—including capecitabine, cyclophosphamide, and aspirin—with regorafenib may increase the proportion of patients with metastatic CRC who benefit from regorafenib without additional toxicity [81]. In contrast, the addition of aspirin to chemotherapy in patients with high-risk rectal cancer did not demonstrate significant clinical benefit (NCT03170115) [82]. Finally, anthelmintic agents such as niclosamide and nitazoxanide are still in early development, and published results are limited.

Collectively, pharmacological agents repurposed to target cancer cell metabolism—such as metformin, statins, and anti-inflammatory compounds—have exhibited promising activity, although clinical outcomes remain heterogeneous. The extent to which their therapeutic efficacy depends on the modulation of metabolic pathways within cancer cells is still uncertain. Moreover, most available evidence is preliminary, underscoring the need for large-scale, rigorously designed clinical trials to substantiate their therapeutic relevance in CRC.

## 5. Discussion and Conclusions

Metabolic reprogramming, a hallmark of cancer, plays a central role in CRC progression and has emerged as a promising therapeutic target, with several metabolic inhibitors currently under investigation. Within this framework, drug repositioning represents an especially attractive approach, as a growing body of evidence indicates that drugs originally developed for metabolic, cardiovascular, inflammatory, psychiatric, or antiparasitic conditions can effectively modulate CRC cell metabolism, inhibit tumor growth, sensitize cancer cells to standard therapies, and enhance anti-tumor immune responses.

Importantly, recent studies have highlighted that metabolic reprogramming in CRC does not occur in isolation but is strongly influenced by the intestinal microbiome, which shapes both local and systemic metabolic environments [83,84]. Microbial metabolites—such as short-chain fatty acids, secondary bile acids, and polyamines—can modulate host energy metabolism, mitochondrial function, and immune responses, thereby affecting tumor progression and therapeutic outcomes [84]. Consequently, variability in microbiome composition may partly explain inter-patient differences in metabolic dependencies and drug sensitivity.

Looking ahead, the integration of drug repositioning with precision oncology holds great promise for advancing CRC treatment. Multi-omics profiling, including genomics, transcriptomics, metabolomics, and proteomics, can help define patient-specific metabolic dependencies, identify predictive biomarkers, and enable tailored repurposing strategies. In parallel, artificial intelligence and systems biology approaches provide powerful tools to uncover hidden drug–metabolism interactions and to design rational combination therapies. Moreover, the convergence of metabolism-targeted approaches with immunotherapies, such as immune checkpoint inhibitors, may unlock synergistic effects capable of overcoming current resistance mechanisms.

Nevertheless, while repurposed drugs offer a safe and cost-effective route to exploit metabolic vulnerabilities in CRC, their broader clinical adoption still faces important challenges, such as potential toxicity at anticancer doses. Indeed, many compounds discussed in this review—such as metformin, statins, and NSAIDs—exhibit favorable safety and cost profiles but often require higher doses to achieve effective intratumoral concentrations. Pharmacokinetic variability, limited tumor selectivity, and unknown interactions with standard chemo- and immunotherapies represent additional major obstacles to clinical translation. Future studies should therefore focus on refining dosing regimens, improving formulation and delivery, and validating predictive biomarkers to enhance efficacy while minimizing adverse effects.

In conclusion, although further mechanistic studies, biomarker validation, and large-scale clinical trials are required, the integration of metabolism-based interventions with drug repositioning has the potential to transform the therapeutic landscape of CRC and ultimately pave the way toward more effective, personalized, and accessible treatments, improving both survival and quality of life for patients worldwide.

## Figures and Tables

**Table 1:** Repurposed drugs targeting metabolic pathways in colorectal cancer (CRC).

Drug Class	Representative Drugs	Main Mechanisms of Action	CRC Models	Refs
**Anti-diabetic drugs**	**Metformin**	↑ AMPK and ↓ mTOR Modulates inflammatory responses and apoptosis in CSCs Regulates miRNAs involved in glycolysis and mitochondrial respiration	In vitro (HCT116, PMF-K014 spheroids) In vivo (Apc^min/+^ mice, sporadic CRC mice) Clinical samples.	[29,30,31,32,33,34,35,36,37,38]
	**Dapagliflozin**	↓ Glycolysis ↑ ERK phosphorylation	In vitro (HCT116) In vivo (MC38 graft model)	[39,40]
	**Sitagliptin**	↓ CD26^+^ cell growth ↓ CD24/CTNNB1/SOX4 axis ↓ MDR1 mRNA, p-AKT, NFκB2 sensitizes to 5-FU	In vitro (CRC stem cells)	[41,42,43]
**Cardiovascular drugs**	**Statins** **(lovastatin, pitavastatin, atorvastatin, simvastatin)**	↑ Mitochondrial dysfunction ↑ AMPK, autophagy and apoptosis ↓ Proinflammatory cytokines ↑ ER stress and autophagy ↓ IGF-1R	In vitro (HCT116, SW480, HT-29) In vivo (obese and Apc^min/+^ mice)	[44,45,46,47,48]
	**Cardiac glycosides (Oleandrin)**	↓ Mitochondrial activity ↑ Apoptosis (↑ caspase-3/9, cytochrome c, BAX ↓ Bcl-2, GSH) ↑ Autophagy	In vitro (SW480, HCT116, RKO, CaCO-2); human tissues (Na^+^/K^+^-ATPase α3 distribution)	[49,50]
	**Beta-blockers (Propranolol, ICI-118,551)**	↑ Mitochondrial dysfunction, cytochrome c release; ↓ Hypoxia adaptation ↓ EGFR-Akt-ERK 1/2	In vitro (HT-29)	[51,52]
**NSAIDs**	**Aspirin**	↓ Glutaminolysis Acetylates G6PD and glycolytic enzymes; ↑ AMPK/NRF2/miR-34 ↓ mTOR ↓ Pyrimidine and arachidonic acid metabolisms; Carnitine shuttle; Modulation of linoleate and glycerophospholipid metabolism	In vitro (HCT116); in vivo (Apc^fl/fl^ mice, clinical biopsies, plasma samples)	[53,54,55,56,57,58]
	**Diclofenac**	↓ c-Myc; ↓ LDH activity; ↓ HSF1, Hsp70/Hsp27; Sensitizes to 5-FU and ionizing radiations	In vitro (LS174T, LoVo), in vivo (xenografts)	[59]
	**Celecoxib**	↓ HK activity, ATP ↑ oxidative stress	In vitro (DLD1, HCT116, HT-29) In vivo (graft model)	[60]
**Antidepressants**	**Fluoxetine**	Induces p53-independent apoptosis (mitochondrial depolarization, calcium overload, ATP depletion); ↓ OxPhos and glycolysis ↓ Xenograft growth, angiogenesis, ↓ mitochondrial complex III and IV	In vitro (CRC cells); in vivo (xenografts)	[61,62,63]
	**Sertraline**	↑ Autophagy (↑ LC3-II/LC3-I, ↓ p62); ↑ ROS; G0/G1 arrest; Sensitizes to 5-FU	In vitro (MC38, CT26, HT-29, LS1034)	[64,65]
	**Citalopram**	Alters CRC metabolism ↓ Proliferation ↑ Apoptosis	In vitro (CaCO-2)	[66,67,68]
	**Vilazodone**	↑ Regorafenib sensitivity via TRIM21-mediated autophagic c-Myc degradation ↓ ENO2, glycolysis, KRAS reprogramming	In vitro (HCT116, SW480) Orthotopic metastatic model Clinical biopsies	[67,68]
**Anthelmintics**	**Niclosamide** **Oxyclozanide**	Mitochondrial uncoupler ↓ ATP; ↑ ROS Autophagic Wnt/β-catenin degradation	In vitro (HCT116, DLD1, CRC240) In vivo (Apc^min/+^, metastatic CRC model)	[69,70,71]
	**Nitazoxanide**	↑ AMPK ↓ AKT/mTOR and Wnt/β-catenin	In vitro (HCT116)	[72]
	**Flubendazole**	↓ mTOR, P62, STAT3, Bcl-2 ↑ Beclin-1, LC3 I/II	In vitro (HCT116, RKO, SW480) In vivo (graft model)	[73,74]
	**Pyrvinium pamoate**	↑ ROS ↓ Akt, p-mTOR, GSK3b	In vitro/in vivo CRC models	[75,76]

**Abbreviations:** 5-FU: 5-fluorouracil; AMPK: AMP-activated protein kinase; ATP: adenosine triphosphate; BAX: Bcl-2 associated X; Bcl-2: B-cell CLL/lymphoma-2; CTNNB1: catenin beta-1; EGFR: epidermal growth factor receptor; ENO2: enolase 2; ER: endoplasmic reticulum; ERK: extracellular signal-regulated kinases; G6PD: glucose-6-phosphate dehydrogenase; GSH: glutathione; GSK3b: glycogen synthase kinase-3 beta; HK: hexokinase; HSF1: heat shock factor 1; HSP70: heat shock protein 70; IGF-1R: insulin-like growth factor 1 receptor; KRAS: kirsten rat sarcoma virus; LDH: lactate dehydrogenase; MDR1: multi-drug resistance 1; mTOR: mammalian target of rapamycin; NF-kB: nuclear factor kappa-light-chain-enhancer of activated B cells; NRF2: nuclear factor erythroid 2-related factor 2; ROS: reactive oxygen species; SOX: SRY-Box transcription factor; STAT3: signal transducer and activator of transcription 3; TRIM21: tripartite motif-containing protein 21; Wnt: Wingless and Integration 1. Upregulation and downregulation are represented by upward and downward arrows, respectively.

**Table 2:** Ongoing and completed clinical trials evaluating repurposed metabolic drugs in colorectal cancer (CRC).

Drug	NCT Number	Study Status	Brief Summary	Study Outcome	Cancer Type/Condition	Phase
**Metformin**	**NCT04033107**	UNKNOWN	High-dose vitamin C combined with metformin in the treatment of malignant tumors.	NO	HCC, PC, GC, CRC	PHASE2
	**NCT03800602**	COMPLETED	Nivolumab and metformin work in treating CRC patients with MSS stage IV.	YES	Colorectal Adenocarcinoma|Metastatic MSS Colorectal Carcinoma	PHASE2
	**NCT03359681**	COMPLETED	Metformin in non-diabetic patients with colon cancer on cell growth, immunological and metabolic changes.	NO	Colon Cancer	PHASE2
	**NCT03047837**	TERMINATED	Low-dose ASA and Metformin in Stage I-III CRC Patients	NO	Tertiary Prevention in Colon Cancer	PHASE2
	**NCT02614339**	UNKNOWN	The effect of adjunctive metformin on recurrence of non-DM Stage II High-risk/III colorectal cancer.	NO	Non-DM Stage II High-risk CRC|Non-DM Stage III CRC	PHASE3
	**NCT02473094**	TERMINATED	Metformin in association to chemotherapy with capecitabine and radiation in the neoadjuvant treatment of locally advanced (T3-4N0M0 or TxN1-2M0) rectal carcinomas.	NO	Rectal Neoplasms|Adenocarcinoma|Carcinoma	PHASE2
	**NCT02437656**	COMPLETED	Metformin in combination with neoadjuvant radiochemotherapy in the treatment of locally advanced rectal cancer.	NO	Rectal Cancer	PHASE2
	**NCT01941953**	COMPLETED	Metformin and Fluorouracil in patients with metastatic CRC who have progressed after Oxaliplatin- and Irinotecan-based chemotherapy.	NO	Metastatic CRC	PHASE2
	**NCT01930864**	UNKNOWN	Metformin combined to irinotecan.	NO	Colorectal Neoplasms|Adenocarcinoma	PHASE2
	**NCT01926769**	TERMINATED	Second-line treatment with Metformin and Chemotherapy (FOLFOX6 or FOFIRI) in the second-line treatment of advanced CRC.	NO	Previously Treated Advanced CRC	PHASE2
	**NCT01632020**	TERMINATED	Effects of short-term oral Metformin therapy on biomarkers for tumor growth in subjects with newly diagnosed colon or rectal adenocarcinoma.	YES	Colorectal Neoplasms	PHASE2
**Simvastatin**	**NCT02161822**	UNKNOWN	Simvastatin combined with capecitabine and radiotherapy in locally advanced rectal cancer patients.	NO	Adenocarcinoma of Rectum	PHASE2
	**NCT02026583**	COMPLETED	Simvastatin plus XELOX and bevacizumab as first-line chemotherapy in metastatic CRC patients.	NO	CRC	PHASE2
	**NCT01238094**	UNKNOWN	Second-line XELIRI/FOLFIRI plus simvastatin compared to XELIRI/FOLFIRI plus placebo.	NO	CRC	PHASE3
	**NCT01190462**	UNKNOWN	Cetuximab together with simvastatin in treating patients with advanced or metastatic CRC.	NO	CRC	PHASE2
	**NCT01110785**	UNKNOWN	Simvastatin plus panitumumab in patients with advanced or metastatic CRC.	NO	CRC	PHASE2
	**NCT00313859**	COMPLETED	Simvastatin plus FOLFIRI (irinotecan, 5-FU, leucovorin) in metastatic CRC patients.	NO	Metastatic CRC	PHASE2
**Aspirin**	**NCT05462613**	RECRUITING	Regorafenib plus metronomic chemotherapies and low-dose aspirin as a two-month induction therapy before chemotherapy initiation in the second-line metastatic colorectal carcinoma.	NO	Metastatic CRC	PHASE2|PHASE3
	**NCT04534218**	COMPLETED	Association of regorafenib with metronomic chemotherapy combining capecitabine, cyclophosphamide and low-dose aspirin, for the treatment of patients with metastatic CRC.	NO	CRC, Metastatic CRC	PHASE2
	**NCT03326791**	ACTIVE_NOT_RECRUITING	Adjuvant treatment with low-dose ASA in patients treated with resection for CRC liver metastases.	NO	CRC Liver metastasis	PHASE2|PHASE3
	**NCT03170115**	TERMINATED	Efficacy and feasibility of aspirin use during chemoradiotherapy for high-risk rectal cancer.	NO	Rectal Cancer, Adenocarcinoma|Locally Advanced Malignant Neoplasm|Chemoradiation	PHASE2
	**NCT02647099**	ACTIVE_NOT_RECRUITING	Adjuvant treatment with low dose aspirin in patients with CRC.	NO	CRC	PHASE3
**Celecoxib**	**NCT06903858**	RECRUITING	Neoadjuvant toripalimab plus celecoxib for dMMR/MSI-H locally advanced CRC.	NO	CRC	PHASE2
	**NCT05933980**	RECRUITING	Toripalimab, celecoxib and regorafenib in the treatment of refractory advanced CRC.	NO	CRC Liver metastasis	PHASE2
	**NCT05731726**	RECRUITING	Chemotherapy and cyclooxygenase inhibitors combined with anti-PD-1 monoclonal antibody in resectable CRC patient with the pMMR/MSS phenotype.	NO	PMMR|MSS|MSI-L|Locally Advanced Rectal Carcinoma	PHASE2
	**NCT05281276**	TERMINATED	Chidamide in combination with celecoxib in patients with advanced metastatic CRC.	NO	Metastatic CRC	PHASE1
	**NCT03645187**	RECRUITING	Celecoxib as an adjuvant therapy to chemotherapy in patients with metastatic CRC.	NO	Colon Cancer Stage	PHASE4
	**NCT03403634**	COMPLETED	Celecoxib, recombinant interferon alfa-2b, and rintatolimod in patients with CRC and liver metastasis.	YES	Metastatic Carcinoma in the Liver|Recurrent Colorectal Carcinoma|Stage IV CRC	PHASE2
	**NCT00608595**	TERMINATED	Celecoxib in patients with early-stage rectal cancer.	NO	CRC	NA
**Niclosamide**	**NCT02687009**	TERMINATED	Niclosamide in patients with colon cancer that are undergoing primary resection of their tumor.	NO	Colon Cancer	ALL
	**NCT02519582**	UNKNOWN	Niclosamide in patients with metachronous or synchronous metastases of CRC.	NO	CRC	ALL
**Nitazoxanide**	**NCT06049901**	RECRUITING	Nitazoxanide in patients with metastatic CRC.	NO	Metastatic CRC	PHASE3

**Abbreviations:** 5-FU: 5-fluorouracil; ASA: acetylsalicylic acid; CRC: colorectal cancer; DM: diabetes mellitus; FOLFIRI: folinic acid, fluorouracil, and irinotecan; FOLFOX: folinic acid, fluorouracil, and oxaliplatin; GC: gastric cancer; HCC: hepatocellular carcinoma; MMR: mismatch repair; MSI-H: high microsatellite instability; MSS: microsatellite stable; PC: pancreatic cancer; PMMR: mismatch repair-proficient; XELOX: xeloda and oxaliplatin; XELIRI: xeloda and irinotecan.

## Data Availability

No new data were created or analyzed in this study. Data sharing is not applicable to this article.

## References

[1] Bray F., Laversanne M., Sung H., Ferlay J., Siegel R.L., Soerjomataram I., Jemal A. (2024). Global cancer statistics 2022: GLOBOCAN estimates of incidence and mortality worldwide for 36 cancers in 185 countries. CA Cancer J. Clin..

[2] Schreuders E.H., Ruco A., Rabeneck L., Schoen R.E., Sung J.J.Y., Young G.P., Kuipers E.J. (2015). Colorectal cancer screening: A global overview of existing programmes. Gut.

[3] Robert C. (2020). A decade of immune-checkpoint inhibitors in cancer therapy. Nat. Commun..

[4] Emambux S., Tachon G., Junca A., Tougeron D. (2018). Results and challenges of immune checkpoint inhibitors in colorectal cancer. Expert Opin. Biol. Ther..

[5] Giannakis M., Mu X.J., Shukla S.A., Qian Z.R., Cohen O., Nishihara R., Bahl S., Cao Y., Amin-Mansour A., Yamauchi M. (2016). Genomic Correlates of Immune-Cell Infiltrates in Colorectal Carcinoma. Cell Rep..

[6] Llosa N.J., Cruise M., Tam A., Wicks E.C., Hechenbleikner E.M., Taube J.M., Blosser R.L., Fan H., Wang H., Luber B.S. (2015). The Vigorous Immune Microenvironment of Microsatellite Instable Colon Cancer Is Balanced by Multiple Counter-Inhibitory Checkpoints. Cancer Discov..

[7] Missiaglia E., Jacobs B., D’Ario G., Di Narzo A.F., Soneson C., Budinska E., Popovici V., Vecchione L., Gerster S., Yan P. (2014). Distal and proximal colon cancers differ in terms of molecular, pathological, and clinical features. Ann. Oncol..

[8] Guinney J., Dienstmann R., Wang X., de Reyniès A., Schlicker A., Soneson C., Marisa L., Roepman P., Nyamundanda G., Angelino P. (2015). The consensus molecular subtypes of colorectal cancer. Nat. Med..

[9] Dienstmann R., Vermeulen L., Guinney J., Kopetz S., Tejpar S., Tabernero J. (2017). Consensus molecular subtypes and the evolution of precision medicine in colorectal cancer. Nat. Rev. Cancer.

[10] Cancer Genome Atlas Network (2012). Comprehensive molecular characterization of human colon and rectal cancer. Nature.

[11] Jang M., Kim S.S., Lee J. (2013). Cancer cell metabolism: Implications for therapeutic targets. Exp. Mol. Med..

[12] Pavlova N.N., Thompson C.B. (2016). The Emerging Hallmarks of Cancer Metabolism. Cell Metab..

[13] Zhong X., He X., Wang Y., Hu Z., Huang H., Zhao S., Wei P., Li D. (2022). Warburg effect in colorectal cancer: The emerging roles in tumor microenvironment and therapeutic implications. J. Hematol. Oncol..

[14] Warburg O. (1956). On the Origin of Cancer Cells. Science (1979).

[15] Tufail M., Jiang C.-H., Li N. (2024). Altered metabolism in cancer: Insights into energy pathways and therapeutic targets. Mol. Cancer.

[16] Vander Heiden M.G., DeBerardinis R.J. (2017). Understanding the Intersections between Metabolism and Cancer Biology. Cell.

[17] Martínez-Reyes I., Chandel N.S. (2020). Mitochondrial TCA cycle metabolites control physiology and disease. Nat. Commun..

[18] Wallace D.C. (2012). Mitochondria and cancer. Nat. Rev. Cancer.

[19] Vodicka P., Vodenkova S., Danesova N., Vodickova L., Zobalova R., Tomasova K., Boukalova S., Berridge M.V., Neuzil J. (2025). Mitochondrial DNA damage, repair, and replacement in cancer. Trends Cancer.

[20] Smith A.L.M., Whitehall J.C., Greaves L.C. (2022). Mitochondrial DNA mutations in ageing and cancer. Mol. Oncol..

[21] Sciacovelli M., Frezza C. (2016). Oncometabolites: Unconventional triggers of oncogenic signalling cascades. Free Radic. Biol. Med..

[22] Isono T., Chano T., Yonese J., Yuasa T. (2016). Therapeutic inhibition of mitochondrial function induces cell death in starvation-resistant renal cell carcinomas. Sci. Rep..

[23] Vasan K., Werner M., Chandel N.S. (2020). Mitochondrial Metabolism as a Target for Cancer Therapy. Cell Metab..

[24] Du H., Xu T., Yu S., Wu S., Zhang J. (2025). Mitochondrial metabolism and cancer therapeutic innovation. Signal Transduct. Target. Ther..

[25] Czechowicz P., Więch-Walów A., Sławski J., Collawn J.F., Bartoszewski R. (2025). Old drugs, new challenges: Reassigning drugs for cancer therapies. Cell Mol. Biol. Lett..

[26] Pushpakom S., Iorio F., Eyers P.A., Escott K.J., Hopper S., Wells A., Doig A., Guilliams T., Latimer J., McName C. (2019). Drug repurposing: Progress, challenges and recommendations. Nat. Rev. Drug Discov..

[27] DiMasi J.A., Feldman L., Seckler A., Wilson A. (2010). Trends in Risks Associated With New Drug Development: Success Rates for Investigational Drugs. Clin. Pharmacol. Ther..

[28] Pillai U.J., Ray A., Maan M., Dutta M. (2023). Repurposing drugs targeting metabolic diseases for cancer therapeutics. Drug Discov. Today.

[29] Misirkic Marjanovic M.S., Vucicevic L.M., Despotovic A.R., Stamenkovic M.M., Janjetovic K.D. (2021). Dual anticancer role of metformin: An old drug regulating AMPK dependent/independent pathways in metabolic, oncogenic/tumorsuppresing and immunity context. Am. J. Cancer Res..

[30] Rocha G.Z., Dias M.M., Ropelle E.R., Osório-Costa F., Rossato F.A., Vercesi A.E., Saad M.J.A., Carvalheira J.B.C. (2011). Metformin Amplifies Chemotherapy-Induced AMPK Activation and Antitumoral Growth. Clin. Cancer Res..

[31] Mogavero A., Maiorana M.V., Zanutto S., Varinelli L., Bozzi F., Belfiore A., Volpi C.C., Gloghini A., Pierotti M.A., Gariboldi M. (2017). Metformin transiently inhibits colorectal cancer cell proliferation as a result of either AMPK activation or increased ROS production. Sci. Rep..

[32] Hirsch H.A., Iliopoulos D., Struhl K. (2013). Metformin inhibits the inflammatory response associated with cellular transformation and cancer stem cell growth. Proc. Natl. Acad. Sci. USA.

[33] Orang A., Marri S., McKinnon R.A., Petersen J., Michael M.Z. (2024). Restricting Colorectal Cancer Cell Metabolism with Metformin: An Integrated Transcriptomics Study. Cancers.

[34] Orang A., Ali S.R., Petersen J., McKinnon R.A., Aloia A.L., Michael M.Z. (2022). A functional screen with metformin identifies microRNAs that regulate metabolism in colorectal cancer cells. Sci. Rep..

[35] Nittayaboon K., Leetanaporn K., Sangkhathat S., Roytrakul S., Navakanitworakul R. (2022). Cytotoxic effect of metformin on butyrate-resistant PMF-K014 colorectal cancer spheroid cells. Biomed. Pharmacother..

[36] Tomimoto A., Endo H., Sugiyama M., Fujisawa T., Hosono K., Takahashi H., Nakajima N., Nagashim Y., Wada K., Nakagama H. (2008). Metformin suppresses intestinal polyp growth in *Apc*
^Min/+^ mice. Cancer Sci..

[37] Hosono K., Endo H., Takahashi H., Sugiyama M., Uchiyama T., Suzuki K., Nozaki Y., Yoneda K., Fujita K., Yoneda M. (2010). Metformin suppresses azoxymethane-induced colorectal aberrant crypt foci by activating AMP-activated protein kinase. Mol. Carcinog..

[38] Saito A., Koinuma K., Kawashima R., Miyato H., Ohzawa H., Horie H., Yamaguchi H., Kawahira H., Mimura T., Kitayama J. (2023). Metformin may improve the outcome of patients with colorectal cancer and type 2 diabetes mellitus partly through effects on neutrophil extracellular traps. BJC Rep..

[39] Nasiri A.R., Rodrigues M.R., Li Z., Leitner B.P., Perry R.J. (2019). SGLT2 inhibition slows tumor growth in mice by reversing hyperinsulinemia. Cancer Metab..

[40] Saito T., Okada S., Yamada E., Shimoda Y., Osaki A., Tagaya Y., Okada J., Yamada M. (2015). Effect of dapagliflozin on colon cancer cell [Rapid Communication]. Endocr. J..

[41] Varela-Calviño R., Rodríguez-Quiroga M., Dias Carvalho P., Martins F., Serra-Roma A., Vázquez-Iglesias L., de la Cadena M.P., Velho S., Cordero O.J. (2021). The mechanism of sitagliptin inhibition of colorectal cancer cell lines’ metastatic functionalities. IUBMB Life.

[42] Shih J.-W., Wu A.T.H., Mokgautsi N., Wei P.-L., Huang Y.-J. (2024). Preclinical Repurposing of Sitagliptin as a Drug Candidate for Colorectal Cancer by Targeting CD24/CTNNB1/SOX4-Centered Signaling Hub. Int. J. Mol. Sci..

[43] Eisa A., Hanafy S.M., Khalil H., Elshal M.F. (2024). Sitagliptin synergizes 5-fluorouracil efficacy in colon cancer cells through MDR1-mediated flux impairment and down regulation of NFκB2 and p-AKT survival proteins. J. Biochem. Mol. Toxicol..

[44] Agarwal B., Bhendwal S., Halmos B., Moss S.F., Ramey W.G., Holt P.R. (1999). Lovastatin augments apoptosis induced by chemotherapeutic agents in colon cancer cells. Clin. Cancer Res..

[45] Huang X., Liang N., Zhang F., Lin W., Ma W. (2024). Lovastatin-Induced Mitochondrial Oxidative Stress Leads to the Release of mtDNA to Promote Apoptosis by Activating cGAS-STING Pathway in Human Colorectal Cancer Cells. Antioxidants.

[46] Yasuda Y., Shimizu M., Shirakami Y., Sakai H., Kubota M., Hata K., Hirose Y., Tsurumi H., Tanaka T., Moriwaki H. (2010). Pitavastatin inhibits azoxymethane-induced colonic preneoplastic lesions in C57BL/KsJ- *db/db* obese mice. Cancer Sci..

[47] Cheng W.-M., Li P.-C., Nguyen M.T.-B., Lin Y.-T., Huang Y.-T., Cheng T.-S., Nguyen T.-H., Tran T.-H., Huang T.-Y., Hoang T.-H. (2025). Repurposing pitavastatin and atorvastatin to overcome chemoresistance of metastatic colorectal cancer under high glucose conditions. Cancer Cell Int..

[48] Jang H.J., Hong E.M., Park S.W., Byun H.W., Koh D.H., Choi M.H., Kae S.H., Lee J. (2016). Statin induces apoptosis of human colon cancer cells and downregulation of insulin-like growth factor 1 receptor via proapoptotic ERK activation. Oncol. Lett..

[49] Pan L., Zhang Y., Zhao W., Zhou X., Wang C., Deng F. (2017). The cardiac glycoside oleandrin induces apoptosis in human colon cancer cells via the mitochondrial pathway. Cancer Chemother. Pharmacol..

[50] Yang P., Cartwright C., Efuet E., Hamilton S.R., Wistuba I.I., Menter D., Addington C., Shureiqi I., Newman R.A. (2014). Cellular location and expression of Na ^+^, K ^+^ -ATPase α subunits affect the anti-proliferative activity of oleandrin. Mol. Carcinog..

[51] Shi C.-S., Kuan F.-C., Chin C.-C., Li J.-M. (2023). Modulation of mitochondrial apoptosis by β2-adrenergic receptor blockage in colorectal cancer after radiotherapy: An in-vivo and in-vitro study. Am. J. Cancer Res..

[52] Barathova M., Grossmannova K., Belvoncikova P., Kubasova V., Simko V., Skubla R., Csaderova L., Pastorek J. (2020). Impairment of Hypoxia-Induced CA IX by Beta-Blocker Propranolol—Impact on Progression and Metastatic Potential of Colorectal Cancer Cells. Int. J. Mol. Sci..

[53] Holt A.K., Najumudeen A.K., Collard T.J., Li H., Millett L.M., Hoskin A.J., Legge D.N., Mortensson E.M.H., Flanagan D.J., Jones N. (2023). Aspirin reprogrammes colorectal cancer cell metabolism and sensitises to glutaminase inhibition. Cancer Metab..

[54] Ai G., Dachineni R., Kumar D.R., Alfonso L.F., Marimuthu S., Bhat G.J. (2016). Aspirin inhibits glucose-6-phosphate dehydrogenase activity in HCT 116 cells through acetylation: Identification of aspirin-acetylated sites. Mol. Med. Rep..

[55] Marimuthu S., Chivukula R.S.V., Alfonso L.F., Moridani M., Hagen F.K., Bhat G.J. (2011). Aspirin acetylates multiple cellular proteins in HCT-116 colon cancer cells: Identification of novel targets. Int. J. Oncol..

[56] Din F.V.N., Valanciute A., Houde V.P., Zibrova D., Green K.A., Sakamoto K., Alessi D.R., Dunlop M.G. (2012). Aspirin Inhibits mTOR Signaling, Activates AMP-Activated Protein Kinase, and Induces Autophagy in Colorectal Cancer Cells. Gastroenterology.

[57] Barry E.L., Fedirko V., Uppal K., Ma C., Liu K., Mott L.A., Peacock J.L., Passarelli M.N., Baron J.A., Jones D.P. (2020). Metabolomics Analysis of Aspirin’s Effects in Human Colon Tissue and Associations with Adenoma Risk. Cancer Prev. Res..

[58] Barry E.L., Fedirko V., Jin Y., Liu K., Mott L.A., Peacock J.L., Passarelli M.N., Baron J.A., Jones D.P. (2022). Plasma Metabolomics Analysis of Aspirin Treatment and Risk of Colorectal Adenomas. Cancer Prev. Res..

[59] Schwab M., Dezfouli A.B., Khosravi M., Alkotub B., Bauer L., Birgani M.J.T., Multhoff G. (2024). The radiation- and chemo-sensitizing capacity of diclofenac can be predicted by a decreased lactate metabolism and stress response. Radiat. Oncol..

[60] Han Y., Chen P., Zhang Y., Lu W., Ding W., Luo Y., Wen S., Xu R., Liu P., Huang P. (2019). Synergy between Auranofin and Celecoxib against Colon Cancer In Vitro and In Vivo through a Novel Redox-Mediated Mechanism. Cancers.

[61] Marcinkute M., Afshinjavid S., Fatokun A.A., Javid F.A. (2019). Fluoxetine selectively induces p53-independent apoptosis in human colorectal cancer cells. Eur. J. Pharmacol..

[62] Kannen V., Hintzsche H., Zanette D.L., Silva W.A., Garcia S.B., Waaga-Gasser A.M., Stopper H. (2012). Antiproliferative Effects of Fluoxetine on Colon Cancer Cells and in a Colonic Carcinogen Mouse Model. PLoS ONE.

[63] Kannen V., Garcia S.B., Silva W.A., Gasser M., Mönch R., Alho E.J.L., Heinsen H., Scholz C.-J., Friedrich M., Heinze K.G. (2015). Oncostatic effects of fluoxetine in experimental colon cancer models. Cell. Signal..

[64] He L., Tian Y., Liu Q., Bao J., Ding R.-B. (2024). Antidepressant Sertraline Synergistically Enhances Paclitaxel Efficacy by Inducing Autophagy in Colorectal Cancer Cells. Molecules.

[65] Gil-Ad I., Zolokov A., Lomnitski L., Taler M., Bar M., Luria D., Ram E., Weizman A. (2008). Evaluation of the potential anti-cancer activity of the antidepressant sertraline in human colon cancer cell lines and in colorectal cancer-xenografted mice. Int. J. Oncol..

[66] Beton-Mysur K., Brożek-Płuska B. (2025). Exploring the Impact of Citalopram on Human Colon Cells: Insights into Antidepressant Action Beyond the Brain. Spectrochim. Acta A Mol. Biomol. Spectrosc..

[67] Liu Y.-X., Wan S., Yang X.-Q., Wang Y., Gan W.-J., Ye W.-L., He X.-S., Chen J.-J., Yang Y., Yang X.-M. (2023). TRIM21 is a druggable target for the treatment of metastatic colorectal cancer through ubiquitination and activation of MST2. Cell Chem. Biol..

[68] Ye W.-L., Huang L., Yang X.-Q., Wan S., Gan W.-J., Yang Y., He X.-S., Liu F., Guo X., Liu Y.-X. (2024). TRIM21 induces selective autophagic degradation of c-Myc and sensitizes regorafenib therapy in colorectal cancer. Proc. Natl. Acad. Sci. USA.

[69] Chen W., Mook R.A., Premont R.T., Wang J. (2018). Niclosamide: Beyond an antihelminthic drug. Cell. Signal..

[70] Wang J., Ren X., Piao H., Zhao S., Osada T., Premont R.T., Mook R.A., Morse M.A., Lyerly H.K., Chen W. (2019). Niclosamide-induced Wnt signaling inhibition in colorectal cancer is mediated by autophagy. Biochem. J..

[71] Alasadi A., Chen M., Swapna G.V.T., Tao H., Guo J., Collantes J., Fadhil N., Montelione G.T., Jin S. (2018). Effect of mitochondrial uncouplers niclosamide ethanolamine (NEN) and oxyclozanide on hepatic metastasis of colon cancer. Cell Death Dis..

[72] Ek F., Blom K., Selvin T., Rudfeldt J., Andersson C., Senkowski W., Brechot C., Nygren P., Larsson R., Jarvius M. (2022). Sorafenib and nitazoxanide disrupt mitochondrial function and inhibit regrowth capacity in three-dimensional models of hepatocellular and colorectal carcinoma. Sci. Rep..

[73] Lin S., Yang L., Yao Y., Xu L., Xiang Y., Zhao H., Wang L., Zuo Z., Huang X., Zhao C. (2019). Flubendazole demonstrates valid antitumor effects by inhibiting STAT3 and activating autophagy. J. Exp. Clin. Cancer Res..

[74] Xing X., Zhou Z., Peng H., Cheng S. (2024). Anticancer role of flubendazole: Effects and molecular mechanisms (Review). Oncol. Lett..

[75] Zheng W., Hu J., Lv Y., Bai B., Shan L., Chen K., Dai S., Zhu H. (2021). Pyrvinium pamoate inhibits cell proliferation through ROS-mediated AKT-dependent signaling pathway in colorectal cancer. Med. Oncol..

[76] Schultz C.W., McCarthy G.A., Nerwal T., Nevler A., DuHadaway J.B., McCoy M.D., Jiang W., Brown S.Z., Goetz A., Jain A. (2021). The FDA-Approved Anthelmintic Pyrvinium Pamoate Inhibits Pancreatic Cancer Cells in Nutrient-Depleted Conditions by Targeting the Mitochondria. Mol. Cancer Ther..

[77] Lin N.-Y., Tsai K.-Y., Huang Y.-L., Jong B.-K., Yu Z.-H., Cheng C.-C., Huang S.-H., You J.-F., Lai I.-L. (2025). Metformin’s impact on tumor regression grade in diabetic patients with rectal cancer undergoing neoadjuvant chemoradiotherapy. Sci. Rep..

[78] Kim Y., Kim T.W., Han S.W., Ahn J.B., Kim S.T., Lee J., Park J.O., Park Y.S., Lim H.Y., Kang W.K. (2019). A Single Arm, Phase II Study of Simvastatin Plus XELOX and Bevacizumab as First-Line Chemotherapy in Metastatic Colorectal Cancer Patients. Cancer Res. Treat..

[79] Baas J.M., Krens L.L., ten Tije A.J., Erdkamp F., van Wezel T., Morreau H., Gelderblom H., Guchelaar H.J. (2015). Safety and efficacy of the addition of simvastatin to cetuximab in previously treated KRAS mutant metastatic colorectal cancer patients. Investig. New Drugs.

[80] Lee J., Jung K.H., Park Y.S., Ahn J.B., Shin S.J., Im S.-A., Oh D.Y., Shin D.B., Kim T.W., Lee N. (2009). Simvastatin plus irinotecan, 5-fluorouracil, and leucovorin (FOLFIRI) as first-line chemotherapy in metastatic colorectal patients: A multicenter phase II study. Cancer Chemother. Pharmacol..

[81] Borg C., Klajer E., El Kaddissi A., Ghiringhelli F., Kim S., Vernerey D., Henriques J., Nguyen T., Meurisse A., Fratte S. (2023). P-281 Safety analysis and preliminary clinical results of REPROGRAM-01 phase II study evaluating regorafenib in combination with a multimodal metronomic chemotherapy in patients with metastatic colorectal cancer. Ann. Oncol..

[82] Souza J.C.E.S.O.D., Araujo R., Valadão M., Carrara C., Barbosa M.Á., Guimarães R., Carvalho J., Kovaleski G., Small I., Marins A. (2018). Induction chemotherapy plus chemoradiotherapy with or without aspirin in high risk rectal cancer (ICAR). Ann. Oncol..

[83] Nenkov M., Ma Y., Gaßler N., Chen Y. (2021). Metabolic Reprogramming of Colorectal Cancer Cells and the Microenvironment: Implication for Therapy. Int. J. Mol. Sci..

[84] Cao Q., Yang M., Chen M. (2025). Metabolic interactions: How gut microbial metabolites influence colorectal cancer. Front. Microbiol..

